# Predicting Computed Tomography Scan-Determined Elevated Intracranial Pressure by Ultrasonographic Measurement of Optic Nerve Sheath Diameter: A Prospective Observational Study

**DOI:** 10.7759/cureus.97848

**Published:** 2025-11-26

**Authors:** Kriti Kriti, Asif Ahmed, Sujeet Joshi, Mahajyoti Chakravorty

**Affiliations:** 1 Anaesthesiology, Mandakini Institute of Medical Sciences and Hospital, Aistala, IND; 2 Critical Care Medicine, Tata Main Hospital, Jamshedpur, IND

**Keywords:** computed tomography, intracranial pressure, optic nerve sheath diameter, traumatic brain injury, ultrasound

## Abstract

Introduction

Traumatic brain injury (TBI) is a major public health problem and is associated with short- and long-term adverse clinical outcomes, including disability and death. Neuroimaging with computed tomography (CT) of the brain is used to detect evidence of increased intracranial pressure (ICP) in settings where invasive ICP monitoring is not practiced. Transorbital ultrasound (US) to measure the optic nerve sheath diameter (ONSD) is a non-invasive method for detecting elevated intracranial pressure (EICP). The present study was undertaken to assess the utility of transorbital US to measure ONSD and predict EICP in individuals with moderate-to-severe TBI compared with computed tomography (CT)-detected EICP.

Materials and methods

This prospective observational study was conducted at the Department of Critical Care Medicine in a tertiary care hospital in eastern India. This study prospectively recruited 110 patients aged ≥ 18 years with moderate-to-severe TBI who were admitted to the critical care unit (CCU) between July 1, 2021, and December 31, 2022. EICP was defined by ONSD ≥5.5 mm.

Results

A total of 110 patients with moderate-to-severe TBI were recruited during the study period. The recruited patients were divided into two groups based on the CT evidence of EICP: 1. CT positive (CTP) group: patients with evidence of EICP on CT scan; and 2. CT negative (CTN) group: patients with no evidence of EICP on CT imaging. A CT scan of the brain detected EICP in 58% of the patients (n = 64). An increased ONSD (≥ 5.5 mm) by transorbital US was noted in 69% (n = 76) of the cohort, and a CT brain characteristic of EICP was present in 71% (n = 54). Using the ONSD assessment, 10 patients with positive CT brain features of EICP were missing. Of the patients with moderate-to-severe TBI, a significantly higher proportion of patients with CT-detected EICP had ONSD measurement ≥ 5.5 mm compared with the CTN group. Furthermore, the mean ONSD of the CTP group (5.57 mm) was significantly greater than that of the CTN group (5.20 mm). The CTN group exhibited a significantly greater survival rate, 68.5% (n = 44) of the CTP group and 37% (n = 17) of the CTN group died before discharge. ONSD with a cut-off value of ≥ 5.5 mm had a sensitivity of 84% and a specificity of 65% with positive predictive value and negative predictive value of 70% and 69%, respectively, for predicting CT-determined EICP.

Conclusion

Our study demonstrated that measurement of ONSD by transorbital ultrasound is a sensitive tool for predicting EICP in patients with moderate-to-severe TBI in the critical care unit. CT evidence of EICP is a risk factor for reduced survival in patients with moderate-to-severe traumatic brain injury.

## Introduction

Traumatic brain injury (TBI) is a major public health problem and is associated with short- and long-term adverse clinical outcomes, including disability and death. India has the highest rate of TBI in the world, with over 1 million people suffering from TBI [1]. As per the World Health Organization (WHO) estimates, nearly 90% of deaths due to injuries occur in low- and middle-income countries (LAMICs), which contribute 85% of the world’s population [2,3].

According to the Monro-Kellie doctrine, elevated intracranial pressure (EICP) can result from any mechanism that increases the volume of one or more components of the brain, i.e., blood, brain tissue, or cerebrospinal fluid (CSF). The addition of a fourth component, such as mass, edema, or hemorrhage, can also increase intracranial pressure (ICP). Elevated intracranial pressure is defined as an intracranial pressure of more than 20 mm of mercury (Hg) and is a devastating complication of TBI [4]. Beyond the compensatory limit, elevation of ICP leads to cerebral ischemia and brain herniation.

Although intraventricular catheter measurement of ICP, an invasive method, is considered the "gold standard" of ICP measurement and diagnosis of EICP, it is associated with the risk of central nervous system infection and intracranial hemorrhage. Furthermore, it is not readily available in LAMICs. Noninvasive methods of measuring EICP are useful in these settings for diagnosis and subsequent monitoring.

Neuroimaging with computed tomography (CT) of the brain is used to detect evidence of increased ICP in institutions where invasive ICP monitoring is not practiced. However, a CT scan carries a high risk of transport to an imaging unit, and it is not always possible to reimage to detect any subsequent change in ICP.

Transorbital ultrasound (US) to measure the optic nerve sheath diameter (ONSD) is a noninvasive method to detect EICP. It has the potential to become a useful screening tool with transorbital US. The utility of noninvasive ICP assessment is being recognized, especially in resource-limited settings where invasive monitoring may not be possible. Therefore, rapid diagnosis of EICP with judicious use of this noninvasive monitoring method can play a key role in the successful management of patients with TBI.

Several studies have examined the use of ONSD to predict increased intracranial pressure in different groups of patients. Various studies have used varying cut-offs of ONSD to detect increased ICP. The primary goal of the study is to determine the diagnostic accuracy of ONSD measurement by US for detecting CT-defined EICP in individuals with moderate-to-severe TBI compared with CT-detected EICP. This paper was previously presented as a poster at the CRITICARE 2024 conference held in Kolkata on March 2, 2024.

## Materials and methods

This prospective observational study was conducted at the Department of Critical Care Medicine, Tata Main Hospital, Jamshedpur, a tertiary care hospital in eastern India. The study was initiated after the Institutional Ethics Committee, Tata Main Hospital, Jamshedpur, India, approved the study (TMH/AC/IEC/JUN/065/2021, dated June 23, 2021). This study prospectively recruited patients aged ≥18 years with moderate-to-severe TBI (score of Glasgow Coma Scale <13 on initial assessment) who were admitted to the critical care unit (CCU) between July 1, 2021, and December 31, 2022 [5]. Patients under the age of 18 years, those with ocular trauma affecting the orbital structure and/or eyeball, those with ocular pathology (glaucoma, lens opacity) affecting the optic nerve and/or orbital cavity, and those with no cranial CT scan performed within the first six hours after admission were excluded from the study.

Baseline demographic data, Glasgow Coma Scale (GCS) score at initial assessment, quick Sequential Organ Failure Assessment (qSOFA), features suggestive of EICP on CT head performed within six hours of admission, requirement for vasoactive drugs, blood gas parameters at the time of admission, length of stay in the CCU, and outcomes (survival and death before discharge from the hospital) were recorded for all patients along with the ONSD measurement within three hours of CT brain imaging [6]. A CT brain scan was performed on a Siemens 32-Slice machine (SOMATOM Scope, Shanghai, China). The CT film or report was not disclosed before the ONSD measurement. EICP by CT was defined by the presence of any of the following: midline shift ≥5 mm, bilateral cisternal or sulcal effacement, mass effect, collapse of ventricles, and gross edema leading to loss of grey white matter differentiation [4,7].

Measurement of optical nerve sheath diameter

An experienced neuroanesthesiologist first validated the ONSD measurement by the investigators using 25 US measurements. Ocular ultrasonography was performed using a high-frequency linear transducer, 6-13 MHz of M-Turbo, Sonosite Fujifilm, on patients who met the inclusion criteria after admission to the CCU following a brain CT scan. A generous amount of jelly was applied to the closed eyes, and ONSD was measured 3 mm posterior to the globe in the transverse plane in each eye, while the patient lay supine in a resting condition with 30° head elevation. With a total of three measurements from each eye, the average of both eyes was calculated for comparison. EICP was defined by ONSD ≥5.5 mm [8].

Statistical analysis

For statistical analysis, data were entered into a Microsoft Excel 2016 (Microsoft Corp., Redmond, WA, USA) spreadsheet and analyzed using SPSS (version 27.0; SPSS Inc., Chicago, IL, USA). Data were summarized as means and standard deviations for numerical variables and counts and percentages for categorical variables. The Z-test (standard normal deviate) was used to test for significant differences in proportions. Comparison between CT criteria for EICP according to continuous variables was assessed using the Mann-Whitney U test. Comparison between CT criteria for EICP according to categorical variables was evaluated using the chi-square test or Fisher’s exact test, as appropriate. Comparison between ONSD and continuous variables was assessed using the Mann-Whitney U test. Comparison between ONSD and categorical variables was evaluated using the chi-square test or Fisher’s exact test, as appropriate. The diagnostic value of ONSD for detecting EICP determined by CT criteria was assessed using the ROC curve. All tests were one-sided. A p-value <0.05 was considered significant.

## Results

A total of 120 moderate-to-severe TBI patients who were to undergo brain CT were prospectively enrolled in the study, of which 10 patients were excluded because they did not meet the inclusion criteria. Of 110 patients, eight had moderate TBI, and 102 had severe TBI. ONSD was measured within three hours of the CT scan in the CCU. The recruited patients were divided into two groups based on the CT evidence of EICP: 1. CT positive (CTP) group: patients with evidence of EICP on CT scan; and 2. CT negative (CTN) group: patients with no evidence of EICP on CT imaging. Table 1 demonstrates the cohort baseline characteristics. Significant cerebral edema was the most common CT finding in our cohort, followed by midline shift, mass effect, sulci effacement, and ventricular collapse.

**Table 1:** Baseline characteristics of the study cohort SD: standard deviation; n: number, RTA: road traffic accident; qSOFA: quick Sequential Organ Failure Assessment; ONSD: optic nerve sheath diameter; GCS: Glasgow Coma Scale; CT: computed tomography; EICP: elevated intracranial pressure.

Characteristics	Total
Age (mean, SD)	39.85 (15.10)
Gender (n, %) female	14 (12.7)
Gender (n, %) male	96 (87.3)
Mode of injury, RTA (n, %)	96 (87.3)
Mode of injury, fall (n, %)	8 (7.3)
Mode of injury, assault (n, %)	6 (5.5)
qSOFA (mean, SD)	1.42 (0.65)
GCS (mean, SD)	5.00 (2.09)
ONSD (mean, SD)	5.41 (0.52)
Vasoactive drugs (n, %)	26 (23.6)
Ventilator support (n, %)	104 (94.5)
Midline shift (n, %)	22 (20.0)
Sulci effacement (n, %)	8 (7.3)
Mass effect (n, %)	16 (14.5)
Ventricular collapse (n, %)	4 (3.6)
Significant edema (n, %)	32 (29.1)
CT EICP (n, %)	64 (58.2)
ONSD ≥ 5.5 mm (n, %)	76 (69.1%)

Table 2 provides a comparison of the baseline characteristics for the CTP and CTN groups. The age and gender of the two study groups did not significantly differ from one another. The mean quick Sequential Organ Failure Assessment (qSOFA) score was less than 2 in both groups; therefore, even though qSOFA was significantly higher in the CTP group, it is unlikely to have any clinical value. Furthermore, because both groups’ mean GCS scores were below 9, a low score may not have clinical significance even though it may be statistically significant. CT scan of the brain detected EICP in 58% of the patients (n = 64). An increased ONSD (≥5.5 mm) by transorbital sonography was noted in 69% (n = 76) of the cohort, and a CT brain characteristic of EICP was present in 71% (n = 54). Using the ONSD assessment, 10 patients with positive CT brain features of EICP were missing. Of the patients with moderate-to-severe TBI, a significantly higher proportion of patients with CT-detected EICP had ONSD measurement ≥5.5 mm compared with the CTN group. Furthermore, the mean ONSD of the CT-detected EICP group was significantly greater than that of the CTN group. Patients with moderate and severe TBI had mean ONSD values of 5.1 mm (standard deviation (SD), 0.25) and 5.4 mm (SD = 1), respectively.

**Table 2:** Baseline characteristics (CTP and CTN groups) CTP: computed tomography positive; CTN: computed tomography negative; χ²: Pearson chi-square test; t: Independent sample t test; SD: standard deviation; n: number; RTA: road traffic accident; qSOFA: quick Sequential Organ Failure Assessment; GCS: Glasgow Coma Scale; ONSD: optic nerve sheath diameter. Bold value indicates the statistical significance of p < 0.05.

Characteristics	CTP (n = 64)	CTN (n = 46)	χ²/t value	p value
Age (mean, SD)	42.19 (16.89)	36.59 (11.58)	2.06ᵗ	0.100
Gender (n, %) male	58 (90.6)	38 (82.6)	1.55χ²	0.253
Gender (n, %) female	6 (9.4)	8 (17.4)		
Mode of injury, RTA (n, %)	58 (90.6)	38 (82.6)	1.94χ²	0.346
Mode of injury, fall (n, %)	4 (6.3)	4 (8.7)		
Mode of injury, assault (n, %)	2 (3.1)	4 (8.7)		
qSOFA (mean, SD)	1.56 (0.71)	1.22 (0.51)	2.96ᵗ	0.004
GCS (mean, SD)	4.91 (1.87)	5.80 (2.27)	2.20ᵗ	0.027
ONSD (mean, SD)	5.57 (0.44)	5.20 (0.56)	3.70ᵗ	<0.00001
ONSD ≥ 5.5 mm (n, %)	54 (84.4)	22 (47.8)	16.74χ²	<0.0001

Of the enrolled patients (n = 110), 44.5% (n = 49) survived to be discharged. Although it was not statistically significant, there was a trend toward longer hospital stays in the CTP group, with a mean hospital stay of 10.19 days compared with 8.9 days in the CTN group. Our study observed that CT evidence of elevated ICP is a risk factor for reduced survival in patients with moderate-to-severe traumatic brain injury. The CTN group exhibited a significantly greater survival rate, 68.5% (n = 44) of the CTP group and 37% (n = 17) of the CTN group died before discharge. Comparison between ONSD-positive (ONSDP, ONSD ≥ 5.5 mm) and ONSD-negative (ONSDN, ONSD < 5.5 mm) groups is displayed in Table 3.

**Table 3:** Baseline characteristics (ONSDP and ONSDN groups) ONSDP: optic nerve sheath diameter positive; ONSDN: optic nerve sheath diameter negative; SD: standard deviation; n: number; RTA: road traffic accident; qSOFA: quick Sequential Organ Failure Assessment; GCS: Glasgow Coma Scale; ONSD: optic nerve sheath diameter; CT: computed tomography; EICP: elevated intracranial pressure. Bold value indicates the statistical significance of p < 0.05.

Characteristics	ONSDP (n = 76)	ONSDN (n = 34)	χ²/t value	p value
Age (mean, SD)	41.95 (15.50)	35.15 (13.19)	2.36ᵗ	0.033
Gender (n, %) male	66 (86.8)	30 (88.2)	0.041χ²	>0.9999
Gender (n, %) female	10 (13.2)	4 (11.8)		
Mode of injury, RTA (n, %)	72 (94.7)	24 (70.6)	16.34χ²	<0.0001
Mode of injury, fall (n, %)	4 (5.3)	4 (11.8)		
Mode of injury, assault (n, %)	0 (0.0)	6 (17.6)		
qSOFA (mean, SD)	1.25 (0.46)	1.43 (0.67)	0.68ᵗ	0.087
GCS (mean, SD)	4.78 (1.85)	6.41 (2.15)	3.84ᵗ	<0.0001
ONSD (mean, SD)	5.57 (0.44)	5.20 (0.56)	2.84ᵗ	<0.00001
CT EICP (n, %)	54 (58.2)	10(29.4)	16.74χ²	<0.05

Sixty-nine percent (n = 76) of the recruited patients had ONSD ≥5.5 mm (ONSDP). In the ONSDP group, 54 patients had CT brain characteristics of EICP. Compared with the patients in the ONSDN group, patients with moderate-to-severe TBI who had elevated ONSD were significantly older. However, a mean age difference of seven years may not be clinically significant in day-to-day practice. In the ONSDP group, a significantly greater proportion of patients had moderate-to-severe TBI resulting from a road traffic accident (RTA). There were no variations in the ONSD measurements for moderate-to-severe traumatic brain injuries resulting from falls and assaults. The relatively small number of assault/fall cases in our cohort may be the reason for this. The ONSDP group had a significantly higher proportion of patients with CT-determined EICP.

**Figure 1:**
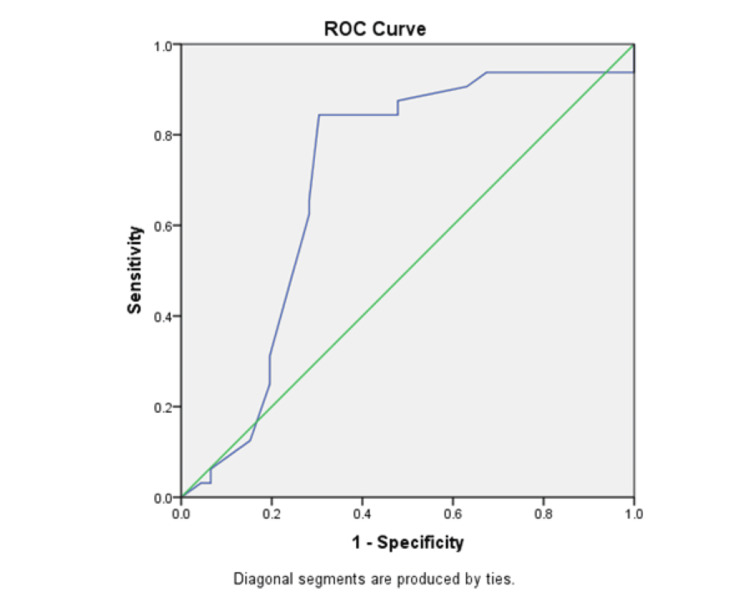
ROC curve The green line indicates the line of no discrimination, and the blue line indicates the actual performance curve. ROC: receiver operating characteristic.

Receiver operating characteristic (ROC) curve analysis of ONSD and CT findings to predict EICP as determined by CT criteria (Table 4). ROC curves of the mean ONSD were made, and the area under the curve (AUC) was measured to determine their diagnostic ability for predicting CT-determined EICP (Figure 1). ONSD measurement by transorbital ultrasound to accurately predict CT-determined EICP with a cut-off value of 5.5 mm had a sensitivity of 84% and a specificity of 65% with positive predictive value (PPV) and negative predictive value (NPV) of 70.0% and 69.0%, respectively, with a significant AUC of 0.826 (p < 0.001).

**Table 4:** Receiver operating characteristic analysis of ONSD ONSD: optic nerve sheath diameter; AUC: area under the curve; CI: confidence interval; PPV: positive predictive value; NPV: negative predictive value.

Test variables	Cut-off value	AUC	95% CI	p value	Sensitivity (%)	Specificity (%)	PPV	NPV
ONSD	5.5	0.70	(0.593, 0.812)	<0.0001	84.4	65.0	70.0	69.0

## Discussion

Prospectively, 110 patients with moderate-to-severe traumatic brain injury (TBI) were enrolled in this study. Various studies have shown that ONSD measurement by US is a sensitive and specific bedside tool for assessing EICP. Our study demonstrated a sensitivity of 84% and specificity of 65%, with PPV and NPV of 70.0% and 69.0%, respectively, in predicting a CT-determined EICP using a cut-off value of ≥5.5 mm for ONSD. Our study demonstrated that patients with moderate-to-severe TBI and CT evidence of elevated ICP had an increased risk of death during the hospital stay.

CCU admissions are common in patients with moderate-to-severe traumatic brain injuries. Identifying EICP, which has been associated with a considerable risk of mortality and morbidity if appropriate therapy is not commenced, can be challenging for each of these patients. CT scan is utilized to identify EICP when invasive monitoring is not available. Notwithstanding the relatively short turnaround time and lack of invasiveness, this neuroimaging method exposes patients to radiation, necessitates high-risk transportation to the imaging unit, and may be difficult to access in LAMICs. Studies have shown that, compared with CT-determined EICP and invasive ICP monitoring, the measurement of the optic nerve sheath diameter (ONSD) by US may be able to diagnose EICP with varying sensitivity and specificity.

With the added benefit of avoiding high-risk transport, ONSD measurement by point-of-care ultrasound (POCUS) is achievable without interfering with ongoing therapy, particularly if serial monitoring and treatment response are to be evaluated. Transorbital ultrasound measurement of ONSD at the bedside is a quick, secure, patient-friendly, and easily teachable method that is also highly reproducible and has good interobserver reliability [9]. Since invasive ICP monitoring is not performed in our unit, we used neuroimaging with cranial CT scanning to identify EICP in our investigation. Hansen and Helmke concluded the utility of sonographic measurements in 54 human optic nerve specimens before and after exposure to pressure [10].

Patients suspected of having EICP due to potential localized intracranial pathology participated in a six-month prospective blinded observational study conducted in the emergency department (ED) by Blaivas and his colleagues [7]. The ONSD measurement by transorbital US accurately predicts the presence of EICP, according to the authors’ premise. Individuals who were suspected of having EICP because of potential focal intracranial pathology were enrolled. An ONSD >5 mm on US was considered abnormal. On CT imaging, 40% of the patients (n = 14) demonstrated evidence of EICP. Our investigation demonstrated that 58% of the patients had EICP identified by brain CT. Variations in the enrolled population may account for the higher proportion of CT-detected EICP. While Blaivas et al. recruited individuals suspected of having EICP due to both trauma and non-trauma causes, the patients in our study had moderate-to-severe TBI [7]. Transorbital US correctly predicted all cases of CT-determined EICP (n = 14) based on ONSD >5 mm by US [7]. In contrast, ONSD could accurately predict EICP in 84% (n = 54) of patients in the CTP group in our study. However, the cut-off to detect EICP in our study was 5.5 mm, whereas Blaivas et al. used 5 mm [7]. The patients without CT evidence of an EICP (CTN group) had a mean ONSD of 4.42 mm (95% confidence interval: 4.15 to 4.72); the group whose EICP was established by CT had a mean ONSD of 6.27 mm (95% confidence interval: 5.6 to 6.89). The mean ONSD of the CTP group in our study was 5.57 mm, whereas that of the CTN group was 5.2 mm. The ONSD demonstrated 100% sensitivity and 95% specificity in detecting CT-determined EICP, respectively, coupled with 93% positive and 100% negative predictive values [7]. In contrast, our study’s sensitivity and specificity were lower than those of Blaivas et al., given that we employed different cut-off values to predict CT-determined EICP. The small sample size, the one-time assessment conducted in the ED, and the absence of follow-up measurements were some of the study’s drawbacks. Nonetheless, the research established a close correlation between increased ONSD by transorbital ultrasonography and CT-determined EICP [7].

Munawar et al. conducted a prospective observational study and enrolled 100 patients who were admitted to the CCU due to TBI and ranged in age from 18 to 65 years [11]. Diffuse cerebral edema on CT scan was reported in 49% of the cohort, similar to our study, where 54% of our cohort had CT evidence of EICP, and it correlated well with increased ONSD as measured by bedside transorbital US. In contrast to our study’s 5.7-mm mean ONSD, the CT-detectable EICP group’s ONSD was 6.1 mm. The ONSD with a cut-off value of 5.8 mm has a sensitivity of 94% (95% confidence interval: 84.05% to 98.79%) and a specificity of 96% (95% confidence interval: 86.7% to 99.52%). The PPV was 92.08% (95% confidence interval: 86.28% to 98.96%), whereas the NPV was 94.23% (95% confidence interval: 84.47% to 98.00%) [11].

Major and his colleagues conducted a study to assess whether ONSD measured by the US could correctly predict the presence of EICP [12]. The authors performed a three-month prospective observational study in adults using the US to measure ONSD in patients who underwent CT neuroimaging from the ED. A mean ONSD value of 5 mm was taken as the cut-off value to diagnose EICP. They enrolled 26 patients in the study. Of them, only 26% (n = 7) of patients had evidence of EICP on a CT scan, compared with 58% in our study. The sensitivity and specificity of the ONSD measurement in predicting EICP were 86% (95% confidence interval: 42% to 99%) and 100% (95% confidence interval: 79% to 100%), respectively. An ONSD value of more than 5 mm was 100% specific (95% confidence interval: 76% to 100%) and 60% sensitive (95% confidence interval: 27% to 86%) to detect any acute intracranial abnormality. Although the cut-off to predict CT-detected EICP was different from that in our study, the sensitivity was similar. In our study, the sensitivity and specificity for detecting a CT-determined EICP using a cut-off value of 5.5 mm for ONSD were 84.4% and 65%, respectively.

Malayeri et al. conducted a study to identify raised ICP in children and compared transorbital sonographic measurement of ONSD by US in CT/US-identified raised ICP with a control group of normal ICP in children [13]. The mean ± standard deviation (SD) ages of the cohort were 6.9 ± 5.6 years and 6.8 ± 5.5 years in the case and control groups, respectively. ONSD measured by US was significantly greater in children with EICP compared with the control group, and the mean ONSD was 5.6 ± 0.6 mm (range: ± 0.7 to 7.6 ± 0.6 mm) in the case group compared to 3.3 ± 0.6 mm (range: 2 ± 0.6 to 4.35 ± 0.6 mm) in the control group. The study also reported a significant difference in the means between the two groups.

Kerscher et al. published a study on a mixed cohort of pediatric neurosurgical patients, who underwent invasive ICP measurement (via intraparenchymal ICP probe, 29%; closed extraventricular drainage, 7%, or puncture of the shunt reservoir, 15.3%, the lumbar CSF space, 8.4%) in both sedated and awake children, with only a minority being ICU patients [14]. Seventy-two children were enrolled. They reported a good correlation between ONSD and ICP (r = 0.52, p < 0.01) in their cohort. Children older than one year had a better correlation (r = 0.63; p <0.01), whereas those younger than one year had a poor correlation (r = 0.21). Infants with open fontanelles did not correlate at all. The best ONSD cut-off values to identify ICP ≥ 15 and ≥ 20 mmHg were 5.28 mm and 5.57 mm, respectively, for the entire cohort [14].

Soldatos et al. evaluated the predictability and usefulness of transorbital sonography of the optic nerve to diagnose EICP and compared noninvasive ONSD measurement with an invasive intraparenchymal catheter to determine a raised ICP [15]. The authors investigated the correlation between ONSD measurements and simultaneous noninvasive and invasive ICP measurements in moderately to severely brain-injured adults. For this study, 76 critical care patients were recruited. Of these, 50 patients had moderate-to-severe brain injuries. Twenty-six cases with no intracranial pathology were used as controls. The ONSD measurement by the US and estimated ICP by transcranial Doppler sonography were both significantly increased (6.1 ± 0.7 mm and 26.2 ± 8.7 mmHg, respectively; p < 0.0001) in patients who suffered a severe brain injury compared with patients who had a moderate brain injury (4.2 ± 1.2 mm and 12.0 ± 3.6 mmHg) and compared with control individuals (3.6 ± 0.6 mm and 10.3 ± 3.1 mmHg). The mean ONSD in our study cohort was 5.4 mm, that of patients with severe TBI was 5.4 mm, and that of patients with moderate ONSD was 5.1 mm. For patients with severe brain injury, the ONSD measurements had a strong correlation with estimated ICP values (r = 0.80, p < 0.0001). In patients with severe brain injury, ONSD measurements correlated with invasive ICP values (r = 0.68, p = 0.002). They reported that the best cut-off value of the ONSD for predicting EICP was 5.7 mm with a sensitivity of 74.1% and specificity of 100% [15]. The authors concluded that there is a strong correlation between ONSD measurements, noninvasive and invasive measurements of ICP, and CT brain findings in brain-injured adults. Our study cohort had 92% (n = 102) of patients who sustained a severe head injury, and only 8% (n = 8) suffered from moderate TBI. Soldatos et al. demonstrated the predictability of ONSD in detecting raised ICP using both a noninvasive method (transcranial Doppler sonography) and an invasive ICP measurement [15]. In contrast, our study demonstrated the predictability of ONSD in detecting CT-determined EICP. The sensitivity of predicting EICP was 74.1% with a cut-off value of 5.7 mm ONSD, whereas our study demonstrated similar sensitivity in predicting EICP with a 5.5 mm cut-off.

Altayar et al. investigated the utility of ONSD measurement by the US for the diagnosis of EICP determined by CT criteria, as well as invasive ICP measurement in adult patients with TBI [16]. ICP greater than 22 mmHg was considered as EICP. They recruited 48 patients for the study. Fifty-eight percent (n = 28) of the patients had evidence of EICP on CT. Our study population had a proportion similar to that of CT-determined EICP. The mean value of ONSD was greater in the CTP group (6.3 ± 0.6 mm) compared with 5.5 ± 0. 7 mm in the CT-negative group. Our study demonstrated a similar finding; however, the mean ONSD of 5.57 mm in the CTP group and 5.2 mm in the CTN group was slightly smaller than that in the quoted study. A total of 22 patients with an intraventricular device were subjected to invasive ICP measurement. Of them, 13 patients were found to have EICP. The mean value of the ONSD was 6.6 ± 0.5 mm in the EICP group compared with 5.8 ± 0.8 mm in the normal group, with a significant difference (p = 0.004). ONSD with a cut-off value > 6.1 mm correctly predicted a high ICP with a sensitivity and specificity of 84.62% and 66.67%, respectively. Our study also observed a similar trend in predicting CT-diagnosed EICP, with a sensitivity and specificity of 85% and 65%, respectively. The authors concluded that transorbital sonographic ONSD measurement can be used as a screening tool for the diagnosis of EICP in adult patients with traumatic brain injury [16].

Sitanaya et al. conducted a cross-sectional study in the ED on 69 patients with the first onset of intracranial pathology [17]. The mean ONSD in the CTP and CTN groups was 0.63 ± 0.06 and 0.57 ± 0.06 cm, respectively. The optimal cut-off value to predict CT-determined EICP was 5.8 mm based on ROC curve analysis [17].

Kim et al. performed a systematic review to verify the diagnostic accuracy of ONSD by the US for detecting EICP by brain CT [18]. They reviewed six studies with 352 subjects to determine the ONSD cut-off for diagnosing CT-detected EICP. The ONSD > 5.0 mm had a pooled sensitivity of 99% (95% confidence interval: 96 to 100) and a specificity of 73% (95% confidence interval: 65 to 80) for detecting CT-determined EICP. The area under the summary receiver operating characteristic curve was 0.981, demonstrating a high degree of accuracy. The authors concluded that a US ONSD > 5.0 mm can be used as a screening tool to identify EICP in adult patients coming to EDs and CCUs. Nevertheless, they recommended that more meta-analyses based on patient-level databases is necessary to validate these findings [18]. Our cut-off value (5.5 mm) differs from that of the meta-analysis, but both follow the same pattern of greater sensitivity and decreased specificity, highlighting the importance of this tool for screening diagnostics [18].

Robba et al. included seven prospective studies with 320 adult subjects over the previous three decades in their meta-analysis to evaluate the diagnostic accuracy of transorbital sonographic ONSD measurements for the assessment of EICP [19]. The meta-analysis only included studies with invasive ICP monitoring. Two studies used a cut-off value of 25 mmHg to diagnose EICP, and five of the included studies used a cut-off ICP value of 20 mmHg. They noted that ultrasonographic ONSD may be a helpful method for detecting EICP, particularly in settings where invasive ICP monitoring is not feasible. While the specificity of our study, which was 65%, was slightly lower than the specificity of seven studies, which ranged from 74% to 96%, the sensitivity of the ONSD by the US compared with the invasive ICP measurement for the detection of EICP ranged from 88% to 94% in the seven different studies that are comparable with our study [19].

Limitations

There are several limitations to our study. The results of this single-center observational study, which included patients with moderate-to-severe traumatic brain injury, cannot be extrapolated to other patient populations. The gold standard for ICP measurement is invasive ICP monitoring, although our study did not employ invasive techniques to detect EICP, which would have yielded a more precise comparison. We identified elevated ICP using brain CT. The additional drawbacks were the small sample size, the one-time evaluation with ONSD measurements, the lack of follow-up assessments with ONSD measurements, and potential confounders such as hemodynamic instability or pre-existing neurological conditions.

## Conclusions

Our study demonstrated that measurement of ONSD by transorbital ultrasound is a sensitive tool for detecting EICP in patients diagnosed with moderate-to-severe traumatic brain injuries in the critical care unit, particularly in a setting where an invasive method of ICP measurement is not available. Patients with moderate-to-severe TBI who had CT evidence of EICP are likely to have an increased risk of adverse outcomes in the form of increased mortality. There was a trend toward increased mortality with ONSD ≥ 5.5 mm, but this was not statistically significant.

## References

[1] Taylor CA, Bell JM, Breiding MJ, Xu L (2017). Traumatic brain injury-related emergency department visits, hospitalizations, and deaths: United States, 2007 and 2013. MMWR Surveill Summ.

[2] Murray CJ, Lopez AD (1996). Global Health Statistics: A Compendium of Incidence Prevalence and Mortality Estimates for Over 200 Conditions. Global Health Statistics: A Compendium of Incidence Prevalence and Mortality Estimates for Over 200 Conditions.

[3] Murray CJ, Lopez AD (1996). The Global Burden of Disease: A Comprehensive Assessment of Mortality and Disability from Diseases, Injuries and Risk Factors in 1990 and Projected to 2020.

[4] Fernando SM, Tran A, Cheng W (2019). Diagnosis of elevated intracranial pressure in critically ill adults: systematic review and meta-analysis. BMJ.

[5] Teasdale G, Jennett B (1974). Assessment of coma and impaired consciousness: a practical scale. Lancet.

[6] Singer M, Deutschman CS, Seymer CW (2016). The Third International Consensus Definitions for sepsis and septic shock (sepsis-3). JAMA.

[7] Blaivas M, Theodoro D, Sierzenski PR (2003). Elevated intracranial pressure detected by bedside emergency ultrasonography of the optic nerve sheath. Acad Emerg Med.

[8] Amini A, Kariman H, Arhami Dolatabadi A (2013). Use of the sonographic diameter of optic nerve sheath to estimate intracranial pressure. Am J Emerg Med.

[9] Ballantyne SA, O'Neill G, Hamilton R, Hollman AS (2002). Observer variation in the sonographic measurement of optic nerve sheath diameter in normal adults. Eur J Ultrasound.

[10] Hansen HC, Helmke K (1996). The subarachnoid space surrounding the optic nerves: an ultrasound study of the optic nerve sheath. Surg Radiol Anat.

[11] Munawar K, Khan MT, Hussain SW, Qadeer A, Shad ZS, Bano S, Abdullah A (2019). Optic nerve sheath diameter correlation with elevated intracranial pressure determined via ultrasound. Cureus.

[12] Major R, Girling S, Boyle A (2011). Ultrasound measurement of optic nerve sheath diameter in patients with a clinical suspicion of raised intracranial pressure. Emerg Med J.

[13] Malayeri AA, Bavarian S, Mehdizadeh M (2005). Sonographic evaluation of optic nerve diameter in children with raised intracranial pressure. J Ultrasound Med.

[14] Kerscher SR, Schöni D, Hurth H, Neunhoeffer F, Haas-Lude K, Wolff M, Schuhmann MU (2020). The relation of optic nerve sheath diameter (ONSD) and intracranial pressure (ICP) in pediatric neurosurgery practice: part I: correlations, age-dependency and cut-off values. Childs Nerv Syst.

[15] Soldatos T, Karakitsos D, Chatzimichail K, Papathanasiou M, Gouliamos A, Karabinis A (2008). Optic nerve sonography in the diagnostic evaluation of adult brain injury. Crit Care.

[16] Altayar AS, Abouelela AZ, Abdelshafey EE (2021). Optic nerve sheath diameter by ultrasound is a good screening tool for high intracranial pressure in traumatic brain injury. Ir J Med Sci.

[17] Sitanaya SN, Kamayanti F, Nugroho HA, Prabowo B (2022). Comparing ultrasonographic optic nerve sheath diameter to head computed tomography scan to predict intracranial pressure elevation. SAGE Open Med.

[18] Kim SE, Hong EP, Kim HC, Lee SU, Jeon JP (2019). Ultrasonographic optic nerve sheath diameter to detect increased intracranial pressure in adults: a meta-analysis. Acta Radiol.

[19] Robba C, Santori G, Czosnyka M (2018). Optic nerve sheath diameter measured sonographically as non-invasive estimator of intracranial pressure: a systematic review and meta-analysis. Intensive Care Med.

